# Latent Glanders in the Horse

**Published:** 1883-01

**Authors:** G. W. Bowler


### THE JOURNAL

OF

### COMPARATIVE MEDICINE AND SURGERY.

VOL. IV. JANUARY, 1883. No. 1.

### ORIGINAL COMMUNICATIONS.

### ART. I.—LATENT GLANDERS IN THE HORSE ILLUSTRATED BY THREE CASES.

BY G. W. BOWLER, M.D.V.S.

THE following cases of Glanders will serve to show the length of time the disease may be dormant in the system without giving much outward sign of its presence, and how particular the veterinarian requires to be on making an examination of horses supposed to be affected with Glanders or Chronic Gleet. The first of the cases I have undertaken to report for your JOURNAL was that of a bay gelding, aged, a very fast pacer, owned by a livery stable keeper in Newport, Kentucky. From such information as I could derive in reference to the case it appears that the horse in question had been sick for some months past, and as the owner informed me, had been treated for some imaginary disease of the kidneys, but instead of improving under the treatment had become much worse.

At the time I was called upon to see the horse, the owner

90630

stated that he wished me to make an examination in order to discover the true character of the disease with which he was affected; consequently I gave him a very careful examination.

I found the animal in very poor condition; being low in flesh, an unhealthy appearance of the hair, legs slightly swollen, slight muco purulent discharge from left nostril, some enlargement of left submaxillary gland, which was partly attached to submaxillary bone; whilst the Schneiderian membrane displayed several ugly chancrous ulcers. After this examination I did not hesitate in pronouncing it a case of Chronic Glanders and ordered the animal destroyed, which the owner promised to have done.

I left the stable and scarcely even gave the case a second thought until about twelve months had elapsed, when being called upon to see a patient in Covington, Kentucky, my attention was called to a bay gelding being led to halter, and which had the appearance of a fine, healthy animal. The owner addressed me as follows: "Hello, Doctor! Do you know this horse?" I answered, "no." "Well," said he, "this is the pacing horse, Sam, you pronounced glandered about a year ago." "Indeed," said I, "is that so?" "Yes," said he. "You were slightly mistaken that time, wer'nt you?" I went up to the horse and again examined him, and found it was the same horse I had been called to see a year previous. I answered, "no; I was not mistaken. This horse was glandered when I examined him a year ago and he is a glandered horse to-day." On making this reply he set up a loud laugh at my supposed ignorance, and I left for home wondering at the strangeness of the case.

About two months after this conversation had taken place, I was informed by a gentleman who was present at the time I speak of, that he would like me to come at once and see the horse in question, as the owner was about to have him destroyed. I at once repaired to the stable in which I had before seen him. And what a change had taken place in that short time. Instead of the sleek-haired fine looking horse he before appeared to be, I now found him poor in flesh, with the old discharge from the nostril, the Schneiderian membrane thickened and ulcerated, the body in most parts filled with farcy buds, many of which were discharging, and which gave the animal a most loathsome appearance. I asked the owner if he was now satisfied that my diagnosis was a correct one? He said that he was now well satisfied that such was the case, but that he never would have believed it had he not seen it. The animal was now taken to the commons and shot. Thus we find that in this case the animal had made an apparent recovery, but that the disease had lain dormant in the system for about fourteen months before it again broke forth in its most malignant form.

The next case I shall record was that of a chestnut colt rising five years old, the property of a well known gentlemen in this city, the owner of the celebrated three mile mare, "Black Maria."

This colt was a very promising trotter, his owner having refused $1,000 for him as a three year old, and at the time he was first supposed to be affected with glanders, was about a month after he had recovered from a severe attack of typhoid pneumonia. I was then called in to see him again on account of a slight swelling of the left submaxillary gland supposed to be the result of a cold, but, as the proprietor of the stable had that morning noticed a drop or two of blood issuing from the nostril, he thought it might be more serious than he had supposed. On examination I found the left submaxillary gland considerably tumefied, slight discharge from the nostril and on the membrane I detected two ulcers, while on the back was a farcy bud, and one on the neck. I at once pronounced it a case of glanders and so informed the owner, but instead of having the colt destroyed he insisted on my treating him by way of experiment, to which I at last consented. A stable for the purpose was obtained, and the colt placed in it so that he was entirely isolated from any other stock. I kept him under treatment for six weeks, by which time the ulcers had healed, the discharge ceased, and further than the appearance of the cicatrix where the ulcers had been, nothing appeared to be the matter with the colt. I now turned him over to the owner, but cautioned him that the disease was at any time liable to break out again; he therefore concluded to give him a run on grass where he could do no harm. Just at this time a dealer came along having a black pacing horse he was desirous of selling, and on seeing the colt, which he felt certain had been suffering from an attack of distemper only, offered to trade. An exchange was accordingly made, the owner giving colt, harness, blankets, halters and everything he had previously worn, for the black pacer, and the dealer taking all further responsibilities. In three weeks from this time the chestnut colt was offered for sale at the public stables. He appeared to be in splendid health, and as the auctioneer stated his pedigree, etc., he also, in a sarcastic tone, informed the audience that I had some time ago pronounced him glandered, but that he was no more glandered than he himself. Instead, however, of its having the effect he expected, it had an entirely different result—as not a single offer was made for him and he was returned to the stable. On the following day a gentleman who makes some pretensions to veterinary science and trading in horses purchased him for $150, and a few days afterward drove him up to my office, in a light trotting wagon. He then asked me if it was true that I had pronounced the colt glandered? I told him it was. When he remarked, "Well, I cannot see anything the matter with him." I responded, "Perhaps not, but I can." This ended our interview.

In about a month afterwards the gentleman sold the colt to a prominent dealer in this city, who kept him for eight months when one day his first owner happened to pass through the stable and recognizing him, censured his present owner for keeping the colt amongst the other horses after what had been said. To this the gentleman replied that he didn't want any advice on the subject, as he knew very well that there never had been any glanders about the colt. A few more words passed between them and nothing further was said until two months afterwards when the disease again made its appearance in a most loathsome form, compelling the present owner to return him to his previous one, who had the satisfaction of seeing him destroyed after passing through the hands of three dealers, each of whom considered himself an expert in detecting such diseases.

The third and very important case, was that of the celebrated trotting mare Proteine, whose record almost approached that of the Queen of the Turf, "Maud S." She was owned in Cincinnati and well known in sporting circles.

This valuable mare was first noticed to be wrong, about the beginning of September, 1880. She was then stabled at Chester Park, and commenced to discharge from the left nostril, but it was generally supposed to be a slight cold as she had for some time been rather off her feed. I was called to see her about the 8th of September, when I found her discharging freely from the left nostril, the discharged matter was of a mixed colour and very sticky as it had adhered all the way round the nasal passage which at once excited my suspicions of the case. On proceeding with my examination I discovered two or three chancrous ulcers on the Schneiderian membrane, the left submaxillary gland was considerably tumefied, but not attached to the submaxillary bone, and the general appearance of the mare was not that of an animal in good condition. I informed the owners of my suspicions that the mare was glandered, but it was agreed that I make another examination in the course of a week.

When I next examined her, I proposed to have the mare locked up during the night so that no one could go near her, and that I might see her before her nostrils had been cleansed, which was done.

On this examination I found the discharge from the left nostril much as I found it before, with the exception that it was streaked with blood, and the ulceration had considerably increased and descended. There was no swelling of the legs, or farcy buds on the body. But I was satisfied the mare was glandered, and she was removed during the night and taken to Kentucky.

On account of the celebrity and value of the mare, I proposed to the owners that we demonstrate to their entire satisfaction, by inoculation, that the mare was glandered. This was agreed to by both parties, and I accordingly made the necessary preparations, one of which was the purchase of a horse to carry out the test.

Just as these arrangements had been completed, one of the owners of the mare refused to have the operation performed, as a stable keeper (one of those men to be found in every community who is always ready to cure everything that is incurable) made his appearance and assured the owner that he would cure the mare for a stipulated sum. He now took charge of her and commenced the usual treatment of injecting the nostrils, etc., and at the same time she was allowed to run in a paddock adjoining a pasture in which was another horse owned by a lady in this city. This state of affairs continued for some months, and the mare was pronounced *nearly* cured when all at once the horse in the adjoining pasture commenced swelling in the legs, and, a veterinarian being called in he pronounced the animal suffering from farcy and glanders, and the horse was killed by his orders. The owners of the mare now informed me they were agreed to have the inoculation test, and I was again preparing for it when the same owner upset the business by listening to a number of illiterate persons, I therefore concluded to have nothing further to do with the mare, but to let matters take their own course. A year had now elapsed, since I first saw the mare, when I was informed that she was entirely well and was going into training at once.

She was accordingly brought over to Cincinnati from Kentucky, and all the prominent horsemen, together with two Veterinarians, (one of whom never ceases informing the public that he is an M. R. C. V. S. L. and hangs his diploma in the office of a livery stable in every city or town he visits) were invited to be present. About fifty persons examined the mare and pronounced her all right, as she looked tolerably well, was fleshier than usual, and had altogether ceased discharging from the nostril.

The Veterinarians (?) stated that she had suffered from nasal gleet, but was entirely well. In consequence of this exhibition, a paragraph appeared in the next morning's paper, stating what had occurred, also remarking that a certain Veterinarian (of course referring to me) had pronounced her glandered, but that such was not the case, as the worthies I have before made mention of had entirely cured her, and that the mare now bid fair to be the competitor of Maud S.

On the following day, Mr. Wolfstain, one of the owners, and the gentleman who had always placed confidence in my diagnosis of the case, met me on the street and wished to know my opinion of the mare since the appearance of the paragraph in the daily paper. I stated to him that notwithstanding all that had been said and printed, I had not up to the present time had the slightest occasion to change my opinion of the case. Mr. W. then went to see his partner, and disposed of his interest in the mare for, $1,000.

She was now placed in the hands of a trainer at Piqua, Ohio, who commenced driving her, but found she had no endurance. She was then taken out of his hands and it was decided to make a brood mare of her. She was accordingly sent to the Alexander Stud Farm, Kentucky, and there put to one of the stallions. She was also to remain at this place until she foaled. However, a few weeks only had elapsed before the owner received a dispatch from the Farm, to remove the mare from there at once, and she was consequently returned home the next or same day. It was now concluded to drive her on the road, which was done for a long time until she was WELL. It must be remembered, however, that for some time back the old discharge had made its appearance again.

Mr. Bair, the driver of Maud S, now concluded to take the mare, and place her in the stall previously occupied by Maud S, and it was given out that he was going to beat the time of the celebrated Maud S with this mare. *She is the great comet of the future*, etc., etc.

After Mr. Bair, had been driving the mare for some time, he concluded to give her up, finding that she had neither speed nor endurance. From this time forth she gradually declined. The nasal discharge was ever present, also the enlarged submaxillary gland, and in the glutinous discharge small particles of tuberculous matter could readily be distinguished. This state of affairs continued until the mare had been suffering for nearly two years when she was found to be dying from obstruction of the air passages, and it was at last decided to destroy her. Another convincing proof of her condition ought to have been sufficient for even those who would not allow themselves to believe the mare was glandered, when it was known that a Hambletonian colt owned by the groom having charge of the mare, and who had always kept him with her to show that she was all right, eventually became affected in like manner with her and died from a most loathsome attack of glanders. So much so that it was considered necessary to burn down the stable in which he had been confined in order to get rid of the disease.

